# Self-Knowledge Dim-Out: Stress Impairs Metacognitive Accuracy

**DOI:** 10.1371/journal.pone.0132320

**Published:** 2015-08-07

**Authors:** Gabriel Reyes, Jaime R. Silva, Karina Jaramillo, Lucio Rehbein, Jérôme Sackur

**Affiliations:** 1 Laboratoire de Sciences Cognitives et Psycholinguistique (ENS, CNRS, EHESS), PSL Research University, Paris, France; 2 Université Pierre et Marie Curie, Paris, France; 3 Escuela de Psicología, Universidad Austral de Chile, Valdivia, Chile; 4 Centro de Apego y Regulación Emocional, Universidad del Desarrollo, Santiago, Chile; 5 Departamento de Psicología, Universidad de La Frontera, Temuco, Chile; Technion - Israel Institute of Technology, ISRAEL

## Abstract

Modulation of frontal lobes activity is believed to be an important pathway trough which the hypothalamic-pituitary-adrenal (HPA) axis stress response impacts cognitive and emotional functioning. Here, we investigate the effects of stress on metacognition, which is the ability to monitor and control one's own cognition. As the frontal lobes have been shown to play a critical role in metacognition, we predicted that under activation of the HPA axis, participants should be less accurate in the assessment of their own performances in a perceptual decision task, irrespective of the effect of stress on the first order perceptual decision itself. To test this prediction, we constituted three groups of high, medium and low stress responders based on cortisol concentration in saliva in response to a standardized psycho-social stress challenge (the Trier Social Stress Test). We then assessed the accuracy of participants' confidence judgments in a visual discrimination task. As predicted, we found that high biological reactivity to stress correlates with lower sensitivity in metacognition. In sum, participants under stress know less when they know and when they do not know.

## Introduction

Acute stress is associated with altered cognitive functioning, in particular with respect to decision making [1,2]. Under stress, individuals exhibit less flexible cognitive processing [3] together with altered risk and feedback processing [4,5]. Collectively, these effects suggest that stress taxes executive functions [2,6], and thus they point to the potential impact of stress on the regulation and monitoring of decision processes. Indeed, decision is not only about selecting the right option; it is also about the assessing its appropriateness relative to the circumstances, that is, whether one can be confident or not about one's action. Sound confidence judgments are essential for the decision maker both to make behavioral adjustments and to efficiently cooperate with others [7,8]. Optimal confidence judgments should be calibrated (reflect decision performance in the long run), and sensitive (discriminate correct from incorrect decisions). In this study, we focus on the impact of stress on the sensitivity of confidence judgments, also termed metacognitive accuracy.

The stress response consists in a cascade of mechanisms governed by the Hypothalamic-Pituitary-Adrenocortical (HPA) axis, leading notably to the release of cortisol and catecholamines. Within the brain, these hormones are known to target specifically the prefrontal cortex (PFC) [6,9], thereby altering higher cognitive functions. Of considerable interest regarding metacognition, it has been proposed [10,11] that one of the early effects of acute stress is to dampen activity in regions subserving endogenous attention (dorsolateral and medial PFC), in favor of orienting resources to vigilance and action, that is, to exogenous attention.

While the cognitive mechanisms behind confidence judgments are not fully understood, they certainly involve orienting attention inwards and require flexible processing. Most importantly, it has repeatedly been shown that the rostral and dorsal aspect of the lateral PFC is the neural basis for metacognitive accuracy, where the neuroendocrine impact of stress on the brain is thought to be maximal [12,13,14,15,16,17].

Furthermore, it has recently been shown [18] that cortisol release under acute stress reduces the depth of strategizing in the beauty contest game. In this behavioral economics game, participants are tested on their ability to reason about other participants' reasoning. Under stress, participants seem to have shallower strategic mind reading, and we expected that the same might be true for metacognition, as a form of intra-individual mind reading. Thus, converging neurophysiological and cognitive evidence suggest that stress reactivity, and more precisely cortisol release, should lead to alterations in metacognition.

To test this hypothesis, we first designed a first session during which three groups of participants (high, medium and low responders) were constituted, according to the concentration of cortisol in saliva at the peak of hormonal response to interpersonal stress. In a later second session (12 month after session 1), in order to avoid direct contextual effects of stressors, participants performed a perceptual decision task with confidence judgments. We operationalized metacognitive sensitivity as the extent to which confidence judgments discriminate correct and incorrect responses. We predicted that high responders should have lower scores on this measure. Between session 1 and 2 a pilot exploratory study was run (8 months after session 1 and 4 month before session 2) to evaluate general differences in metacognition according to individual cortisol reactivity to stress. Results and data are available at https://osf.io/6zsap/?view_only=39fb68df24094788b9daffcb0fe5a683

## Materials and Methods

### Session 1: stress screening

120 students (mean age: 20.6, *SD*: 1.5; 58 women) from the Universidad de La Frontera (Chile) participated in the study. These 120 participants are common both for the pilot study, as well as for the main experiment. Participants gave their written informed consent (Declaration of Helsinki). The study at all stages was approved by the ethics committee of Universidad de La Frontera. Participants were compensated the equivalent of about $20 for the 2 hour session. Experimental sessions were scheduled from 2:30 to 6:30 pm. Exclusion criteria were: a body mass index < 18 or > 30 kg/m^2^; receiving medical treatment known to affect the HPA axis; a history of psychiatric or neurological disorders; abnormal vision; smoking; pregnant or lactating women, and women taking oral contraceptives. Participants were asked not to eat or brush their teeth one hour before the session, and not to drink alcohol or play sports the day before.

**Fig 1:**
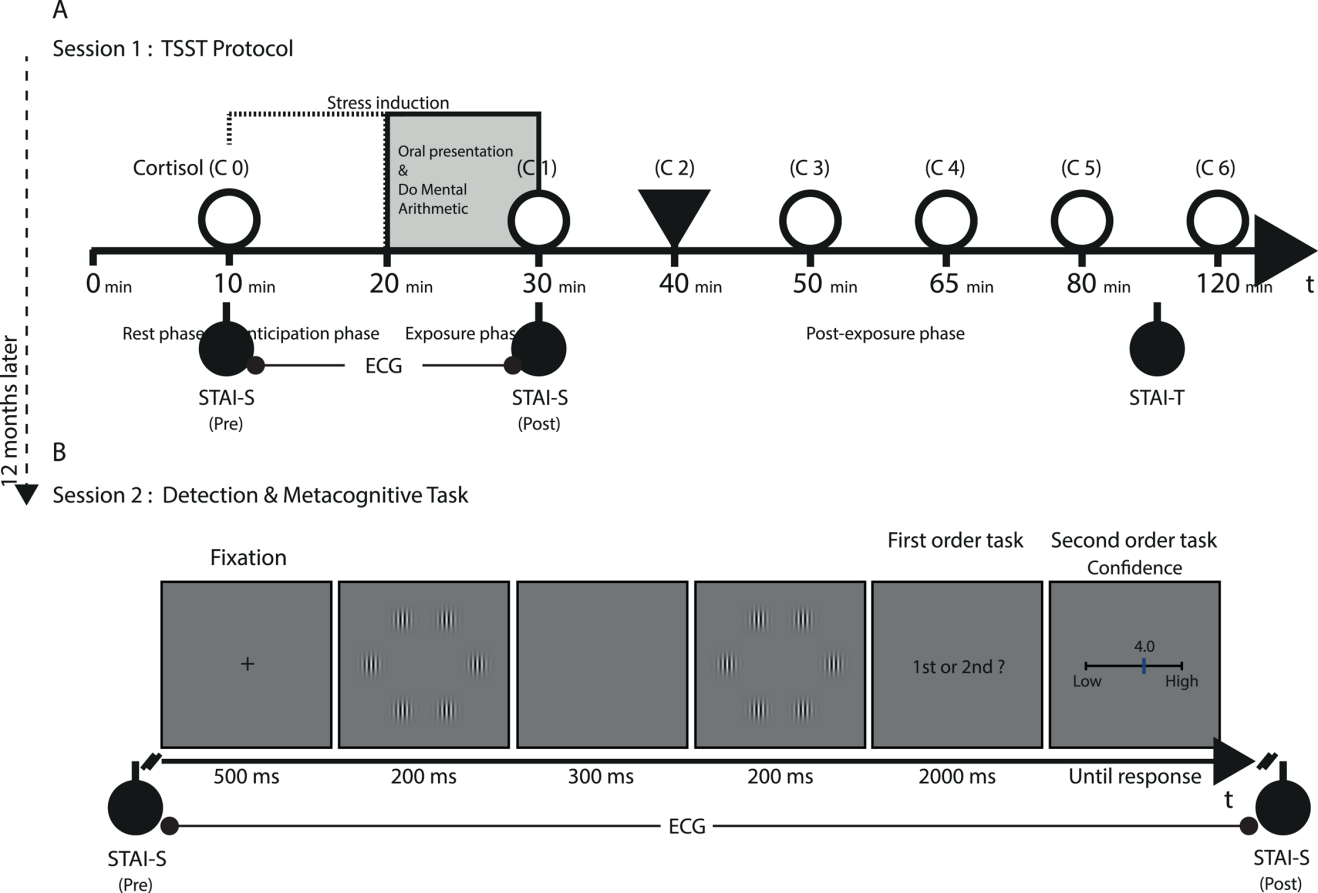
(A) TSST protocol (*session 1*). (B) Detection and Metacognitive tasks (*session 2*).

We applied the Trier Social Stress Test (TSST) [19], with seven interspersed saliva samples to assay cortisol concentration (Fig 1A). The TSST asks that participants perform a ten minutes, videotaped oral presentation and do mental arithmetic in front of a non-supportive panel of three judges. The first saliva sample was taken after a ten minutes rest, immediately followed by the State-Trait Anxiety Inventory (STAI) [20]. Participants then performed the main presentation of the TSST, after which the second saliva sample was taken and participants filled out a second STAI with only the state subscale. Six more saliva samples were taken during a post-exposure rest phase to control normal decrease in the stress reactivity curves. Heart rate (HR) was monitored throughout. Here as well as in the second session, we also recorded electrodermal activity, but technical failures precluded our using the data. Electrodes were located on the medial side of both ankles and the distal anterior aspect of the right forearm (BIOPAC MP150, Goleta, CA). Saliva samples were sent to the Molecular Biology Laboratory of the Universidad de La Frontera, for quantitative determination of cortisol concentration. Salivary concentrations of cortisol were obtained using an enzyme immunoassay commercial kit following the manufacturer’s instructions (DRG Salivary Control ELISA Kit, DRG Instruments GmbH, Germany). Concentrations were obtained by interpolating from a standard curve plotted using the software GraphPad Prism version 5.0 (GraphPad Software, San Diego CA, USA).

### Session 2: confidence accuracy experiment

Based on the results of session 1, we formed three groups of 9 participants with high, medium and low stress reactivity (mean age: 20.1, *SD*: 1.2; 16 women: 5 in the low, 6 in the medium and 5 in the high group), which took part in the experiment one year after session 1. 10 participants who participated in the pilot study were included (3-Low, 3-Medium, 4-High). Participants gave their informed consent and were compensated the equivalent of about $10 for a 1 hour session. Stimuli were arrays of six vertical gabor patches (2.8° in diameter, spatial frequency of 2.2 cycles per visual degree, Fig 1B) on a uniform grey background (luminance: 44.1 cd/m^2^), presented on an imaginary circle (6.2°) at the center of a CRT screen (size 17”, resolution of 1024 X 768 pixels, refresh rate of 100 Hz, viewing distance ~55 cm). Participants were tested in a darkened room.

After a fixation spot (500 *ms*) participants viewed two arrays for 200 *ms* each, separated by an interval of 300 *m*s. In one of the arrays, one random gabor patch had a higher contrast. Participants had to decide in which interval the contrasted gabor was presented (two intervals forced choice task, 2IFC) during a 2000 *ms* response window, by pressing the "Q" (first interval) or "W" (second interval) key on a standard QWERTY keyboard. Participants first went through a calibration stage (120 trials), during which we adapted the target contrast with 1-up 2-down interleaved staircases [21], so as to reach similar accuracy levels for all participants (accuracy at the end of the calibration: 75%, which did not significantly differ from the targeted 71% accuracy). The threshold contrast for each participant was calculated as the average of the last six reversals of both the initially ascending and descending staircases.

During the main experiment, contrast was fixed at the participant's adapted level. Immediately after the 2IFC response, participants were instructed to give the best estimate of their confidence about their decision, by means of a visual analog scale for confidence. We used a half range scale with the labels “*guess*” and “*absolutely certain*” at the left and right ends, which we scored as a probability scale ranging from .5 to 1. The experimental session comprised 320 trials in 8 blocks with a 60 second pause between each block. Before and after the main task participants filled out the STAI-S (state subscale). Heart rate was monitored throughout. As a way to reactivate participants’ stress response, a mild interpersonal stressor in the middle of the task was introduced: Immediately after the fourth block, an error message appeared on screen. The experimenter came into the experimental room with a neutral emotional expression and stated that “*there was a problem with your responses*”, without saying anything about participants’ performance.

## Results

### Session 1

**Fig 2:**
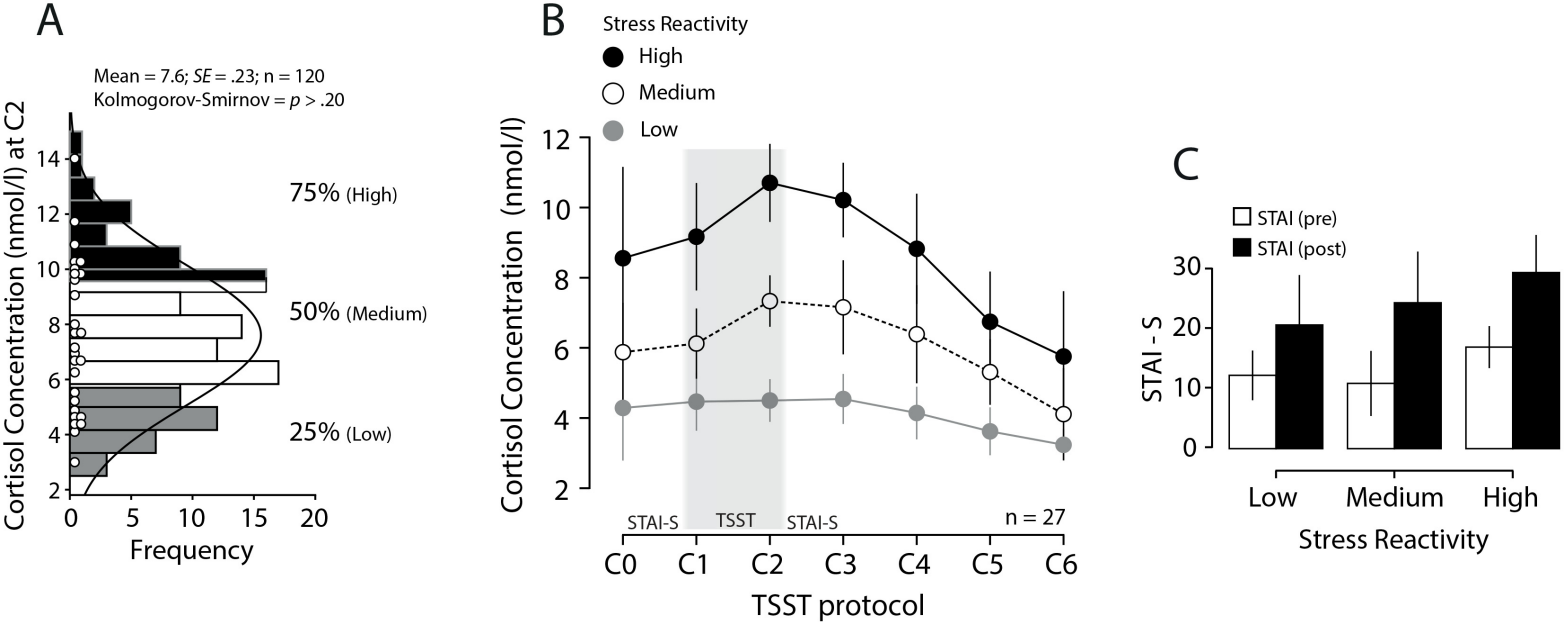
Session 1: Stress screening. A) Histogram of cortisol concentration in saliva at C2, with a superimposed normal fit. Each dot represents a participant tested in session 2. B) Cortisol concentration in saliva during the TSST protocol for each experimental group. Error bars are 95% confidence intervals. C) State-Trait Anxiety Inventory (STAI-S), before and after stress induction. Error bars are 2+/- *SE*.

Cortisol level at C2 (10 minutes after stress induction) was taken as indicative of participants’ reactivity to stress [22]. Participants above the 75th percentile were categorized as having high reactivity (mean: 10.7 nmol/l; *SD*: 1.4; range: 9.6, 14.2; Cortisol increase (C2-C0 or Baseline) = 2.14), participants below the 25th percentile as low reactivity (mean: 4.5 nmol/l; *SD*: .79; range: 2.9, 5.7; Cortisol increase = .21) and participants around the 50th percentile as medium reactivity (mean: 7.3 nmol/l; *SD*: .95; range: 6.3, 9.3; Cortisol increase = 1.45; Fig 2A and 2B). Classifications reached the criteria for distinguishing cortisol responders from non responders (threshold for cortisol responder: 1.1 nmol/l) [23]. We applied the conversion provided by Miller et al. [24], available at http://psylux.psych.tu-dresden.de/i1/biopsych/ac.html for the DRG immunoassay kit.

We randomly selected 9 participants out of each the three above percentile groups. Studies interested in cortisol responders use a similar or smaller numbers of participants (for instance, [25] (20 participants); [26] (20 participants); [27] (20 participants); [28] (26 participants), etc., see many others in [22]). In addition, it is important to note that previous studies select responders and non-responders *ex-post*, which often yield an imbalance between groups. As opposed to that, our selection of responders is done *ex-ante*, ensuring a clean statistical framework for establishing any effect of stress reactivity on the dependent variables.

These groups did not differ with respect to gender (*p >* .73), age (*p >* .50) or stress personality traits (STAI-Trait, *p* > .39). The TSST procedure increased psychological acute stress (Fig 2C): STAI-State was 11.3 point higher after TSST than before (*F*(1,24) = 44.0, *p* < .001, *η* ^*2*^_*p*_ = .65) without difference between groups (*p >* .16) and no interaction (*p >* .46). Mean heart rate (HR) did not differ across groups (*p* > .11), however hear rate variability (HRV), which is indicative of activation of the stress response [29] as computed by standard deviation, was significantly different (*F*(2,24) = 4.33, *p* < .05, *η* ^*2*^_*p*_ = .27). More precisely, collapsing medium and low stress reactivity groups, we found that high reactivity participants had a higher HRV (16.8 beats/min) (low and medium stress participants: 14.0 beats/min; *F*(1,25) = 4.46, *p* < .05, *η* ^*2*^_*p*_ = .15).

### Session 2

#### Stress measures

**Fig 3:**
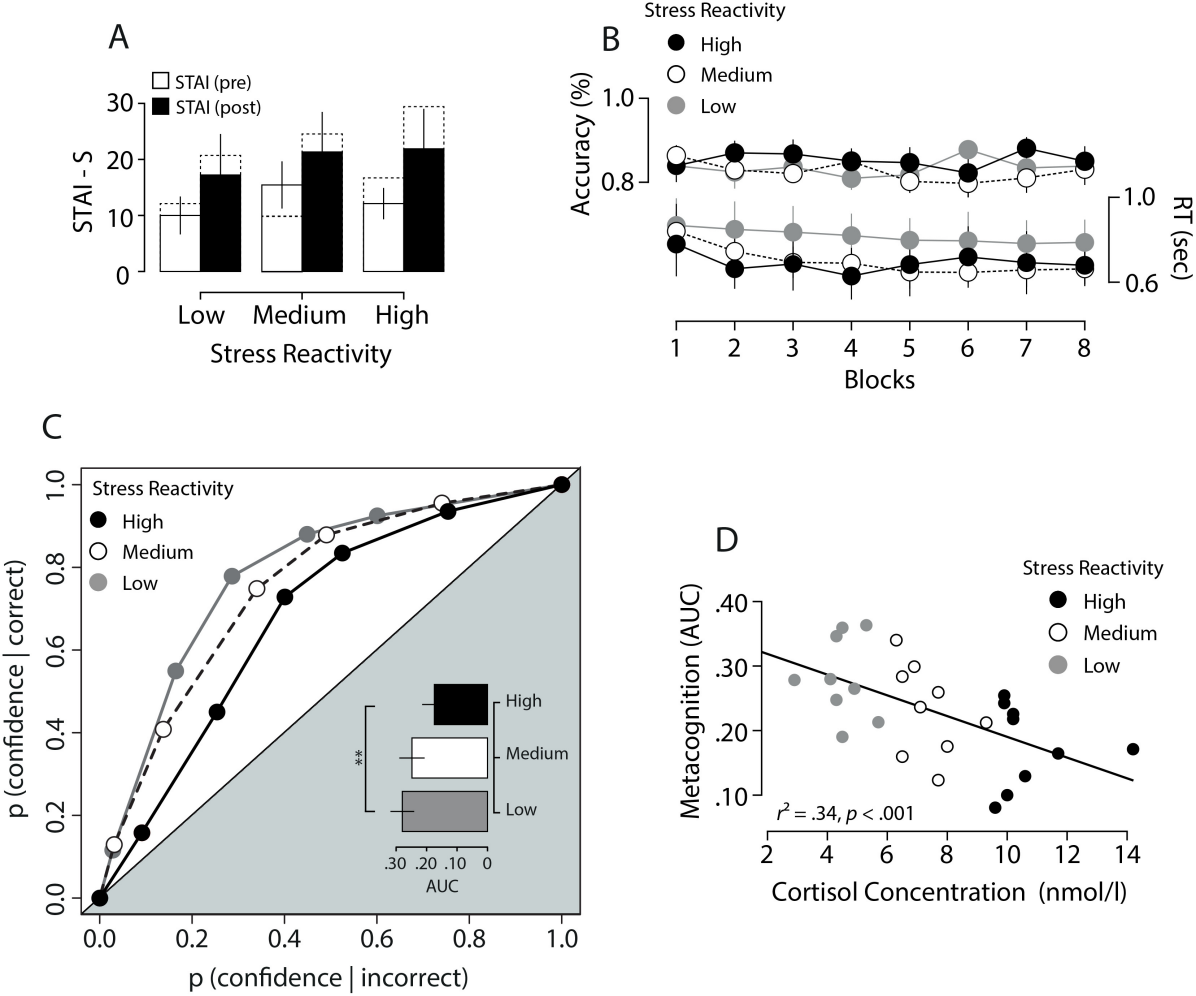
Session 2: Confidence accuracy. A) State-Trait Anxiety Inventory (STAI-S), before and after detection task. Error bars denote 2+/- *SE*. Dashed lines behind each bar represent STAI before and after stress induction during TSST protocol (Session 1). B) Response Time and Accuracy as a function of blocks for each stress group. Error bars denote 95% confidence intervals. C) Metacognitive sensitivity was quantified by area under the type-2 ROC for each stress group. D) Linear regression of AUC scores on cortisol concentration in saliva at C2. Each dot represents one participant.

The experimental context reactivated the stress responses specific to each three groups: Heart rate variability (HRV) was higher during the first half of the experiment for high reactivity participants (7.31 beats/min) than low and medium reactivity participants (6.2 beats/min; *F*(1,25) = 5.78, *p* < .05, *η* ^*2*^_*p*_ = .19). This difference vanished when we took into account the second half of the experiment, but we found a peak of HRV at the time of the interpersonal stressor, only for the high reactivity group (S1 Fig). Psychological stress (STAI-State) increased during the experiment (before / after difference of 7.62 points: *F*(1,24) = 19.1, *p* < .001, *η* ^*2*^_*p*_ = .44; Fig 3A), without difference between groups (*p >* .42) and no interaction (*p >* .65).

#### Performance: accuracy and response times

We excluded trials with response times (RTs) below 200 *ms* and above 2000 *ms* (4.3%). Crucially, there were no differences on first order performance between stress groups: First, the contrast needed to achieve the targeted performance at the end the staircase calibration procedure did not differ across stress groups (*p >* .40). During the main experiment, accuracy increased with respect to the end of the calibration (75% correct), but it was stable at 83% (Fig 3B) and did not differ between groups (*p >* .35) or across blocks (*p >* .93) and these factors did not interact (*p >* .39). RTs were marginally slower for low reactivity participants (*p >* .06; Low: 818 *ms*; Medium: 699 *ms*; High: 692 *ms*) with a significant learning effect (*F*(3.6,88.5) = 5.16, *p* < .01, *η* ^*2*^_*p*_ = .17), but without interaction between these factors (*p >* .23, Fig 3B).

#### Confidence

Mean confidence was .84 and did not differ across stress groups (*p* >.91), meaning first that participants were well calibrated in view of their average performance of 83%, and second, that stress reactivity did not translate into under—or over—confidence. Now, our critical construct was metacognitive sensitivity, or resolution on the confidence scale. We quantified metacognitive sensitivity by the area under the curve (AUC) for the type-2 Receiver Operating Characteristic (ROC), calculated separately for each participant (Fig 3C). This measure consists in plotting one against the other the cumulative proportions of correct and incorrect responses for increasingly liberal confidence percentiles [30]. Type-2 AUC is a model-free, empirical estimate of metacognitive accuracy, which has been used in previous studies [12]. Higher AUC scores denote a stronger link between confidence and accuracy. We relied on this empirical metric for the estimation of metacognitive sensitivity, given that there is currently no consensus on the appropriate signal theoretic models for confidence judgments [31,32].

In order to compute the type-2 ROCs, for each participant we binned the responses on the continuous confidence scale in five equal bins, and we plotted the cumulative proportions of correct against incorrect responses, anchored at 0 and 1 for increasingly liberal bins. We found that higher reactivity was associated with lower AUCs, that is with poorer metacognitive sensitivity (High: .68; Medium: .73; Low: .78). The effect was statistically significant with a one-way ANOVA on AUC with stress reactivity as factor and participants as random effect (*F*(2,26) = 5.86, *p* < .01, *η* ^*2*^ = .33). Post-hoc comparison only showed a difference between low and high reactivity participants (Δ mean = .11, *SE* = .03, *p* < .01, Bonferroni corrected). We next regressed participants' AUCs on cortisol concentrations at C2, and found a significant negative correlation (*r* ^2^ = .34, *β* = -.58, *t*(26) = -3.57, *p* < .001; Fig 3D). Importantly, we checked that AUCs were not related to performance (*p* > .98) or contrast (*p* > .16) or RTs (*p*>.32), see supporting information (S1 and S2 Files) for a more detailed account of RTs. Finally, there was no difference in the response times for confidence between stress reactivity groups (Low: 1258 *ms*; Medium: 1376 *ms*; High: 1165 *ms*, *p >* .56).

## Discussion

We investigated the impact of stress on metacognition. We identified participants with high, medium and low biological reactivity to an interpersonal challenge. High responders were subsequently found to have lower metacognitive accuracy in a perceptual decision task, while no difference was observed on perceptual decision performances or confidence calibration. We insist on the fact that first order performances were equated across participants with an adaptive staircase method, so much so that the decrement in metacognitive sensitivity cannot simply be an indirect consequence of the impact of stress on first order cognition [33]. Thus, even though stress had no effect on first order cognition, and did not create under or over-confidence biases, it decreased metacognitive accuracy. Stress induces a relative blindness to one's cognitive performance.

Contrary to many stress studies, we did not include an explicit stressor prior to the main cognitive task: We relied on the experimental context, combined with individual reactivity, to modulate stress. While this design eliminates contextual cues, it opens the possibility that the observed decrease in metacognitive sensitivity be due to concomitant personality trait differences, rather than to acute stress. However, this interpretation is not favored by our data: First, we selected our three groups based on their cortisol reactivity, and this was not associated with traits differences, as assessed by the STAI-T. Second, the high reactivity group, although defined by peak reactivity at the moment of the interpersonal challenge, already shows an elevated cortisol response at the moment of the first cortisol sample, while it went back to baseline after 2 hours (Fig 2B). Therefore, it seems that for these participants, the mere fact of walking in the laboratory is a stressor, and we may expect that this should be the case at the occasion of the main cognitive task. Indeed, heart rate variability is higher in the high reactivity group during the main task, which gives us a direct sign of stress reactivity. Therefore, it seems more parsimonious to suppose that the impairment in metacognition is due to acute stress activation.

Now, one could argue that the impact of stress reactivity on metacognition is not specific. Indeed, one could argue that the observed effect of stress on metacognition is one among the many effects of stress on executive functioning. While it is clear from the literature that stress does have an impact on executive functions [1,2,3,4,5,6], one should note that in our experiment, metacognitive accuracy is the only outcome variable that shows an effect of stress reactivity. Although we cannot rule out the notion that our perceptual discrimination task is not sensitive enough to elicit such effects, it seems unlikely as first order tasks have consistently demonstrated their higher sensitivity than second order tasks [30]. Thus, if metacognitive decrements were due to a general impairment of cognitive control, it would most probably first be apparent in a change in performance for the first order task.

Now, we see two broad classes of mechanisms through which acute stress would yield such impairment in metacognitive sensitivity: First, it might be that acute stress is associated with intercurrent ruminative thoughts that could interfere with metacognitive processing (“high level” account). These thoughts might potentially be accessible and controlled by participants. Alternatively, it might be that higher circulating cortisol has a direct impact on prefrontal cortex regions that subserve metacognition for decisions (“low level” account). The detrimental effect of stress on metacognition might then be cognitively impenetrable. Note also that cortisol might not be the unique cause of the modifications of prefrontal cortex functioning, as it has been proposed that the cognitive effects of stress are due to the conjoint release of cortisol and catecholamines [6].

Our study adds to the growing literature on dissociations of first and second order performance [12, 31, 32, 34, 35]. Results suggest that first order performance and its subjective evaluation might rely on distinct cognitive mechanisms and information. One promising line of research in this respect would be to study the differential role of working memory in the formation of confidence judgments [34,35]. Indeed, while the first order task is performed immediately after presentation of the stimulus, the second order confidence judgment is performed later and requires the retention of both stimuli and of the participant's own response for a longer time. Thus it might be that stress reactivity affects metacognition through its impact on working memory. If that were the case, we should find that high working memory capacity would protect against stress induced decrease in metacognitive sensitivity, as was recently found for stress induced decrease in model based learning [3].

Additional studies are needed to fully unravel the mechanisms and the generalization of metacognitive impairment under stress. First, the high and low level accounts sketched above, and the working memory hypotheses are not exclusive, and we need to assay their relative weights. Second, recent studies have highlighted the disunity of metacognition [36]. With this in mind, it is of paramount theoretical, clinical and applied importance to determine whether our results hold beyond perceptual decision, that is, whether, for instance, metacognition in general knowledge or memory tasks would similarly be impaired under stress.

## Supporting Information

S1 Fig Heart rate variability during the main experiment. For each participant, we computed the standard deviation of heart rate in non-overlapping windows of 1 minute throughout the duration of the experiment, and smoothed them with locally weighted polynomial regression. The black and red curves are the means of the smoothed heart rate standard deviations for the low and medium reactivity groups on the one hand and for the high reactivity group on the other. Shaded areas are bootstrapped 95% confidence bands for the two groups (5000 samples). Dark blue segments indicate bootstrapped *p* < .05 for the null hypothesis of no difference in heart rate standard deviations between the two groups. Two clusters of significant differences emerge: one at the beginning of the experiment (during the first 8 minutes) and one at the start of the second half of the experiment, just after stress reactivation (gray area).
(EPS)

S1 File Response times on the first order task (2IFC) and mediation analysis. (DOCX)

S2 File Response times on the confidence scale. (DOC)

## References

[1] Lupien SJ, Maheu F, Tu M, Fiocco A, Schramek TE. The effects of Stress and Stress Hormones on human cognition: Implications for the field of brain and cognition. Brain Cogn. 2007;65, 209–237. 17466428 10.1016/j.bandc.2007.02.007

[2] Starcke K, Brand M. Decision making under stress: a selective review. Neurosci. Biobehav. Rev. 2012;36, 1228–1248. 10.1016/j.neubiorev.2012.02.003 22342781

[3] Otto AR, Raio CM, Chiang A, Phelps EA, Daw ND. Working memory capacity protects model-based learning from stress. PNAS. 2013;110, 20941–20946. 10.1073/pnas.1312011110 24324166 PMC3876216

[4] Porcelli A, Delgado M. Acute stress modulates risk taking in financial decision making. Psychol. Sci. 2009;20, 278–283. 10.1111/j.1467-9280.2009.02288.x 19207694 PMC4882097

[5] Schwabe L, Wolf OT. Stress-induced modulation of instrumental behavior: from goal-directed to habitual control of action. Behav. Brain Res. 2011;219, 321–328. 10.1016/j.bbr.2010.12.038 21219935

[6] Arnsten AF. Stress signaling pathways that impair prefrontal cortex structure and function. Nat Rev Neurosci. 2009;10, 410–422. 10.1038/nrn2648 19455173 PMC2907136

[7] Frith CD. The role of metacognition in human social interactions. Phil. Trans. R. Soc. B. 2012;367, 2213–2223‎. 10.1098/rstb.2012.0123 22734064 PMC3385688

[8] Shea N, Boldt A, Bang D, Yeung N, Heyes C, Frith CD. Supra-personal cognitive control and metacognition. Trends Cogn. Sci. 2014;18, 189–196. 10.1016/j.tics.2014.01.006 PMC3989995 24582436

[9] Arnsten AF. Catecholamine modulation of prefrontal cortical cognitive function. Trends Cogn. Sci. 1998;2, 436–447. 21227275 10.1016/s1364-6613(98)01240-6

[10] Qin S, Hermans EJ, Van Marle HJ, Luo J, Fernandez G. Acute psychological stress reduces working memory-related activity in the dorsolateral prefrontal cortex. Biol. Psychiatry. 2010;66, 25–32. 10.1016/j.biopsych.2009.03.006 19403118

[11] Hermans EJ, Henckens MJ, Joëls M, Fernández G. Dynamic adaptation of large-scale brain networks in response to acute stressors. Trends Neurosci. 2014;37, 304–314. 10.1016/j.tins.2014.03.006 24766931

[12] Fleming SM., Weil RS, Nagy Z, Dolan RJ, Rees G. Relating introspective accuracy to individual differences in brain structure. Science. 2010;329, 1541–1543. 10.1126/science.1191883 20847276 PMC3173849

[13] Rounis E, Maniscalco B, Rothwell J, Passingham R, Lau H. Theta-burst transcranial magnetic stimulation to the prefrontal cortex impairs metacognitive visual awareness. Cogn. Neurosci. 2010;1, 165–175. 10.1080/17588921003632529 24168333

[14] Yokoyama O, Miura N, Watanabe J, Takemoto A, Uchida S, Sugiura M, et al Right frontopolar cortex activity correlates with reliability of retrospective rating of confidence in short-term recognition memory performance. Neurosci Res. 2010;68, 199–206. 10.1016/j.neures.2010.07.2041 20688112

[15] Baird B, Smallwood J, Gorgolewski KJ, Margulies DS. Medial and lateral networks in anterior prefrontal cortex support metacognitive ability for memory and perception. J. Neurosci. 2013;33, 16657–16665. 10.1523/JNEUROSCI.0786-13.2013 24133268 PMC6618531

[16] de Martino B, Fleming SM, Garrett N, Dolan RJ. Confidence in value-based choice. Nat. Neurosci. 2013;16, 105–110. 10.1038/nn.3279 23222911 PMC3786394

[17] Fleming SM, Dolan RJ, Frith CD. Metacognition: computation, biology and function. Phil. Trans. R. Soc. B. 2012;367, 1280–1286. 10.1098/rstb.2012.0021 22492746 PMC3318771

[18] Leder J, Häusser JA, Mojzisch A. Stress and strategic decision-making in the beauty contest game. Psychoneuroendocrinology. 2013;38, 1503–151. 10.1016/j.psyneuen.2012.12.016 23312062

[19] Kirschbaum C, Pirke KM, Hellhammer DH. The Trier Social Stress Test: A tool for investigating psychobiological stress responses in a laboratory setting. Neuropsychobiology. 1993;28, 76–81. 8255414 10.1159/000119004

[20] Spielberger CD, Gorsuch RL, Lushene RE. The State-Trait Anxiety Inventory: Test manual. Palo Alto, CA: Consulting Psychologist Press; 1970

[21] García-Pérez MA. Forced-choice staircases with fixed stepsizes: asymptotic and small-sample properties. Vision Res. 1998;38, 1861–1881. 9797963 10.1016/s0042-6989(97)00340-4

[22] Allen AP, Kennedy PJ, Cryan JF, Dinan TG, Clarke G. Biological and psychological markers of stress in humans: focus on the Trier Social Stress Test. Neurosci Biobehav Rev. 2014;38, 94–124. 10.1016/j.neubiorev.2013.11.005 24239854

[23] Miller R, Plessow F, Kirschbaum C, Stalder T. Classification criteria for distinguishing cortisol responders from nonresponders to psychosocial stress: evaluation of salivary cortisol pulse detection in panel designs. Psychosom. Med. 2013;75, 832–840. 10.1097/PSY.0000000000000002 24184845

[24] Miller R, Plessow F, Rauh M, Gröschl M, Kirschbaum C. Comparison of salivary cortisol as measured by different immunoassays and tandem mass spectrometry. Psychoneuroendocrinology. 2013;38, 50–57. 10.1016/j.psyneuen.2012.04.019 22641005

[25] Kirschbaum C, Prüssner JC, Stone AA, Federenko I, Gaab J, Lintz D, et al Persistent high cortisol responses to repeated psychological stress in a subpopulation of healthy men. Psychosom. Med. 1995;57, 468–474. 8552738 10.1097/00006842-199509000-00009

[26] Nater UM, Moor C, Okere U, Stallkamp R, Martin M, Ehlert U, et al Performance on a declarative memory task is better in high than low cortisol responders to psychosocial stress. Psychoneuroendocrinology. 2007;32, 758–763. 17606328 10.1016/j.psyneuen.2007.05.006

[27] Roelofs K, Bakvis P, Hermans EJ, van Pelt J, van Honk J. The effects of social stress and cortisol responses on the preconscious selective attention to social threat. Biol. Psychol. 2007;75, 1–7. 17030399 10.1016/j.biopsycho.2006.09.002

[28] Engert V, Efanov SI, Duchesne A, Vogel S, Corbo V, Pruessner JC. Differentiating anticipatory from reactive cortisol responses to psychosocial stress. Psychoneuroendocrinology. 2013;38, 1328–1337. 10.1016/j.psyneuen.2012.11.018 23246327

[29] Xhyheri B, Manfrini O, Mazzolini M, Pizzi C, Bugiardini R. Heart rate variability today. Prog. Cardiovasc. Dis. 2012;55, 321–331. 10.1016/j.pcad.2012.09.001 23217437

[30] Fleming SM, Lau H. How to measure metacognition. Front. Hum. Neurosci. 2014;8, 1–9. 10.3389/fnhum.2014.00443 25076880 PMC4097944

[31] Scott RB, Dienes Z, Barret AB, Bor D, Seth AK. Blind insight: metacognitive discrimination despite chance task performance. Psychol. Sci. 2014; 25, 2199–2208. 10.1177/0956797614553944 25384551 PMC4263819

[32] Jachs B, Blanco MJ. On the independence of visual awareness and metacognition: a signal detection theoretic analysis. J. Exp. Psychol. 2015;41, 269–276. 10.1037/xhp0000026 25665083

[33] Galvin SJ, Podd JV, Drga V, Whitmore J. Type 2 tasks in the theory of signal detectability: discrimination between correct and incorrect decisions. Psychon. Bull. Rev. 2003;10, 843–876. 15000533 10.3758/bf03196546

[34] Bona S, Cattaneo Z, Vecchi T, Soto D, Silvanto J. Metacognition of visual short-term memory: dissociation between objective and subjective components of VSTM. Front. Psychol. 2013; 4, 1–6. 10.3389/fpsyg.2013.00062 23420570 PMC3572424

[35] Bona S, Silvanto J. Accuracy and confidence of visual short-term memory do not go hand-in-hand: behavioral and neural dissociations. PLoS ONE. 2014;9, 1–10. 10.1371/journal.pone.0090808 PMC3963844 24663094

[36] Fleming SM, Ryu J, Golfinos JG, Blackmon KE. Domain-specific impairment in metacognitive accuracy following anterior prefrontal lesions. Brain. 2014;137, 2811–2822. 10.1093/brain/awu221 25100039 PMC4163038

