# Correction: Self-Knowledge Dim-Out: Stress Impairs Metacognitive Accuracy

**DOI:** 10.1371/journal.pone.0138260

**Published:** 2015-09-10

**Authors:** The PLOS ONE Staff

There is an error in Fig 1. Please see the correct Fig 1 here. The publisher apologizes for the error.

**Fig 1:**
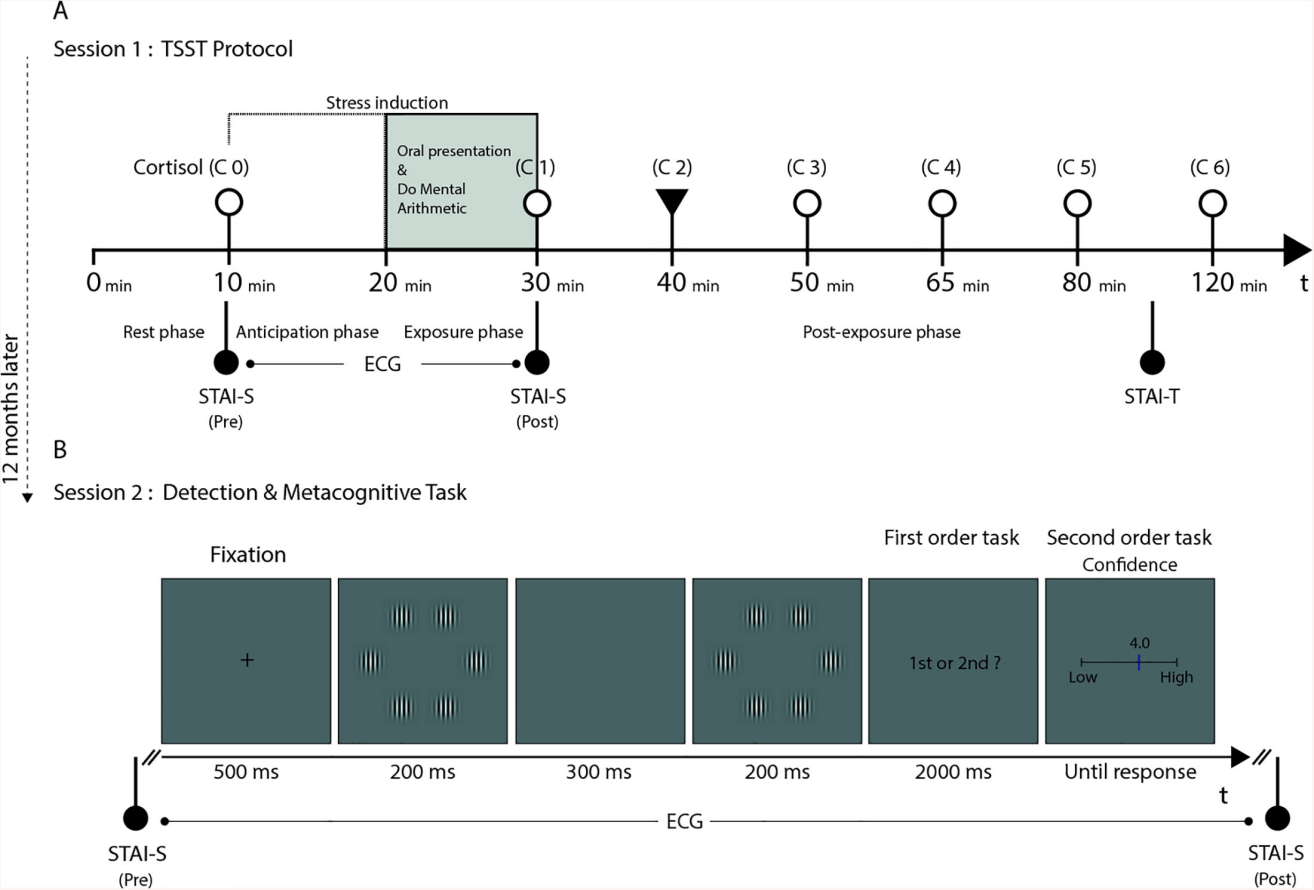
(A) TSST protocol (*session 1*). (B) Detection and Metacognitive tasks (*session 2*).
