# Supplementary material for: Self-Knowledge Dim-Out: Stress Impairs Metacognitive Accuracy
Source: PLoS One. 2015 Aug 7;10(8):e0132320. doi: 10.1371/journal.pone.0132320 (PMC4529147; doi:10.1371/journal.pone.0132320)
Supplement: S1 File — (DOCX) [file pone.0132320.s002.docx]

## Response times on the first order task (2IFC) and mediation analysis

As the only difference on performance on the 2IFC task was that participants in the low stress groups had marginally significant longer response times, we wanted to analyze this trend in greater details with a view to gauge whether response times could in part explain the observed differences in metacognitive accuracy found between stress groups.

First, we analyzed response time distributions as generated by a sequential sampling process (1), so as to so as to extract, for each participant, decision and non decision components of the response. We used the Fast-DM toolbox (2), to fit the diffusion model of Ratcliff (3), for each participant individually, assuming no bias. Notice that since contrast is fixed for each participant, there is only one experimental condition for the set of 320 trials. This yielded the following six parameters for each participant: the drift rate (*v*), the separations of the boundaries (*a*), the non-decision time (*t*), and the variability for each of these. We submitted each of these parameters to separate one-way ANOVAs with stress group as a factor and participant as random effect. The only significant effect was on non-decision times (all other *p*s > .19): non-decision time was longer for the low stress group (409 *ms*) than for the medium (281 *ms*) and high (301 *ms*) stress groups (*F*(2, 24)=4.94, *p* < .05, *η*²=.29). Similarly we found that cortisol release at C2 linearly predicted non-decision time (*β* = -.016, *t*= -2.42, *p* < .05, *r*²=.15). This effect on non-decision time was expected, as accuracy was not different across groups. Difference in any other parameter than non-decision time would in general entail difference in accuracy.

One interesting hypothesis would be that the longer non-decision time for low stress participants would allow them to better encode decision parameters, leading to better confidence accuracy thereafter. Thus, we investigated whether individual differences in non-decision times would mediate the relationship between stress and confidence accuracy. Specifically, we tested the model that the impact of cortisol release at C2 on the area under the type 2 ROC curve (AUC) is mediated by non-decision time. However, we found that, controlling for cortisol, non-decision times did not predicted AUC (*p* > .60), which precludes any further investigation of the mediation.

Thus it seems that stress reactivity is associated both with faster response times and to lower metacognitive accuracy, but that these two effects are mainly independent. Notice further that while the main difference in metacognitive accuracy is between the high group on the one hand and the medium and low groups on the other, differences in response times seems to lie between the low group on the one hand and the high and medium on the other.

**References**

1. Laming DRJ. Information, theory of choice-reaction times. New York: Academic Press; 1968
2. Voss A, Voss J. Fast-dm: A free program for efficient diffusion model analysis. Behav Res Methods. 2007;39, 767–775.
3. Ratcliff RA. A theory of memory retrieval. Psychol. Rev. 1978;85, 59–108.
