# Supplementary material for: Self-Knowledge Dim-Out: Stress Impairs Metacognitive Accuracy
Source: PLoS One. 2015 Aug 7;10(8):e0132320. doi: 10.1371/journal.pone.0132320 (PMC4529147; doi:10.1371/journal.pone.0132320)
Supplement: S2 File — (DOC) [file pone.0132320.s003.doc]

**Response times on the confidence scale**

Due to the response mode (mouse movement and then mouse click) response times on the confidence scale are noisier than on the first order task. Nevertheless, we investigated whether the speed at which participants gave the estimate of their confidence could in part explain their variable metacognitive accuracy.

First, we looked at difference in response times on the confidence scale (hereafter: confidence RT) across stress groups. A one-way ANOVA with group as factor and participant as random effect did not reveal any difference (*p* > .41). Still, when we applied the same diffusion analysis methodology as above to confidence RTs, we found that non-decision times were slower for the low stress group (401 *ms*) than for the medium (290 *ms*) and high (297 *ms*) groups (*F*(2,24)=3.7, *p* < .05, *η*²=.24; all other *p*s > .18). But, again neither confidence RT or non-decision times of confidence RT did predict AUC (*p* > .70), suggesting that the reason why high stress participants have lower metacognitive accuracy is not their hastiness to respond on the confidence scale.
